# Transferability and sustainability of process-based multi-task adaptive cognitive training in community-dwelling older adults with mild cognitive impairment: a randomized controlled trial

**DOI:** 10.1186/s12888-023-04917-3

**Published:** 2023-06-12

**Authors:** Xia Zhao, Caifang Ji, Chen Zhang, Cheng Huang, Yuanyuan Zhou, Lina Wang

**Affiliations:** 1 Department of Geriatric Psychiatry, Suzhou Guangji Hospital, JiangSu, China; 2 School of Medicine, Huzhou University, Zhejiang, China; 3 Department of general medicine, Community health service center of Binhu Street, Zhejiang, China; 4 School of Nursing, Jiangsu College of Nursing, JiangSu, China

**Keywords:** Mild cognitive impairment, Process-based adaptive cognitive training, Executive function, Transfer effects

## Abstract

**Background:**

Cognitive training shows promising effects for improving cognitive domains in individuals with mild cognitive impairment (MCI), including the crucial predictive factor of executive function (EF) for dementia prognosis. Few studies have paid sufficient emphasis on the training-induced effects of cognitive training programs, particularly with regards to targeting EF. A process-based multi-task adaptive cognitive training (P-bM-tACT) program targeting EF is required to examine direct, transfer, and sustainability effects in older adults with MCI.

**Objective:**

This study aimed to evaluate the direct effects of a P-bM-tACT program on EF, the transfer effects on untrained cognitive domains, and further explore the sustainability of training gains for older adults with MCI in the community.

**Methods:**

In a single-blind, randomized controlled trial, 92 participants with MCI were randomly assigned to either the intervention group, participating in a P-bM-tACT program (3 training sessions/week, 60 min/session for 10 weeks) or the wait-list control group, accepting a health education program on MCI (1 education session/ twice a week, 40–60 min/session for 10 weeks). The direct and transfer effects of the P-bM-tACT program were assessed at baseline, immediately after 10 weeks of training, and the 3-month follow-up. Repeated measures analysis of variance and a simple effect test were used to compare the direct and transfer effects over the 3-time points between the two groups.

**Results:**

The P-bM-tACT program yielded a greater benefit of direct and transfer effects in the intervention group participants than in the wait-list control group. Combined with the results of simple effect tests, the direct and transfer effects of participants in the intervention group significantly increased immediately after 10 weeks of training compared to the baseline (F = 14.702 ~ 62.905, p < 0.05), and these effects were maintained at the 3-month follow-up (F = 19.595 ~ 122.22, p < 0.05). Besides, the acceptability of the cognitive training program was established with a high adherence rate of 83.4%.

**Conclusions:**

The P-bM-tACT program exerted positive direct and transfer effects on the improvement of cognitive function, and these effects were sustained for 3 months. The findings provided a viable and potential approach to improving cognitive function in older adults with MCI in the community.

**Trial registration:**

The trial was registered at Chinese Clinical Trials Registry on 09/01/2019 (www.chictr.org.cn; Number Registry: ChiCTR1900020585).

**Supplementary Information:**

The online version contains supplementary material available at 10.1186/s12888-023-04917-3.

## Background

Mild cognitive impairment (MCI) is considered a critical window of opportunity to prevent/intervene and therapeutic intervention of dementia [1]. Executive function (EF) impairment is a frequent and disabling symptom in MCI [2]. EF refers to a set of higher-level cognitive control processes (i.e., working memory, cognitive flexibility, inhibitory control, planning and decision-making) necessary for executing goal-directed complex behaviors [3]. Previous longitudinal studies have indicated that the conversion rate to Alzheimer’s disease (AD) was higher in MCI individuals with lower EF than those with higher EF after 1-year follow-up [4, 5]. Therefore, EF may serve as a crucial predictor of progression from MCI to dementia.

Previous studies suggested that cognitive training is a potentially effective way to improve EF in older adults with MCI [6, 7]. Two recent studies suggest that process-based cognitive training focusing primarily EF is particularly effective among the various training approaches [8, 9]. Process-based cognitive training refers to the repetitive practice of computerized and/or manual cognitive tasks without explicit strategies [8, 9]. Several studies provided evidence to support the effectiveness of process-based cognitive training in enhancing EF among both younger and older individuals [10, 11]. However, the effect of process-based cognitive training targeting EF promotion has not been empirically validated in older adults with MCI.

Additionally, prior studies indicated that the benefits of process-based cognitive training are typically limited to the specific cognitive functions associated with training domains [12, 13]. Given the widespread multi-cognitive functions decline in older adults with MCI, the examination of training-induced cognitive gains on untrained cognitive domains is imperative. Such gains, commonly referred to as transfer effects of training, could be categorized into near transfer effects (i.e., post-training improvement in tasks similar to training tasks) and far transfer effects (i.e., post-training improvement in tasks that assess other functions than those that were trained) [14]. Process-based cognitive training targeting EF has been shown to have positive transfer effects in older adults. Specifically, it could improve not only the targeted EF but also other cognitive domains like memory and attention, as well as everyday activities such as driving and managing finances [15, 16]. However, some studies have suggested that the transfer effects of process-based cognitive training may be limited, as noted by Hill et al. in their systematic review study [17]. As such, more research is needed to better understand the factors that influence the extent and specificity of these effects and to develop more effective and personalized training programs. However, studies on process-based cognitive training with transfer tasks are scarce in older adults with MCI.

Noteworthy, previous studies have shown that adaptive cognitive training, which involves the dynamic adjustment of training task demands based on an individual’s current level of performance, is associated with a more significant transfer effect than non-adaptive [18]. Building on these findings, an adaptive process-based cognitive training program targeting EF in older adults with MCI constitutes a promising tool to explore direct and transfer effects on trained and untrained cognitive domains, as well as sustainability.

To address the above-mentioned issues, we developed a P-bM-tACT program based on our study protocol [19], targeting the improvement of EF in older adults with MCI. The P-bM-tACT program adopted an adaptive principle that gradually changes the difficulty level according to the individual’s capacity to ensure optimal challenge for trainees. Older adults with MCI commonly dwell in the community, yet the absence of symptoms could impede timely diagnosis, thereby causing deferment in suitable management and treatment and heightening the potential for additional cognitive deterioration. Community-based healthcare providers, such as community nurses, play a crucial role in identifying individuals with MCI and providing appropriate care and support. Therefore, the formulation of a nurse-led cognitive training program for individuals with MCI bears auspicious potential for the practice of community healthcare. This study aimed to conduct an empirical study to evaluate the efficacy of the P-bM-tACT program on EF of older adults with MCI and to address three primary research questions.

1. What is the advantage of a P-bM-tACT program in older adults with MCI? We hypothesized that older adults with MCI who participated in the P-bM-tACT program would have a better EF performance.
2. Whether the P-bM-tACT program could engender transfer effects and modify the functions of untrained cognitive domains. We further hypothesized that there were transfer effects in work memory and psychomotor speed would be evoked by the P-bM-tACT program.
3. Whether potential direct and transfer effects related to the P-bM-tACT program could be sustained over time after the short-term intervention. We further hypothesized that all training gains would have sustainability effects.

## Methods

### Study design and ethical consideration

This study was conducted at a community healthcare service center in Huzhou City, China, from March 2019 to March 2022 as a randomized controlled trial with a 3-month follow-up assessment. This study was registered at Clinical Trials.gov ChiCTR1900020585 on 19 January 2019. Results are reported in accordance with the CONSORT Consolidated Standards of Trials statement for non-pharmacological interventions and have obtained ethical approval from the Medical Ethics Committee of the Third People’s Huzhou Hospital of Zhejiang Province (NO.2018-030). All eligible participants voluntarily signed the written informed consent after being fully informed of the study purpose and procedures by the research assistant.

### Study participants

Community-dwelling older adults aged 60 years and over were recruited from community senior centers and healthcare centers in the Wuxing district. Our staff distributed recruitment leaflets, and local healthcare providers also helped to make referrals to our study. Individuals who showed interest were invited for an in-person interview to screen for eligibility by three trained staff. A total of 448 potential individuals who complained of subjective memory impairment were screened in the first eligibility assessments. The second round of eligibility assessments included demographics and health characteristics (medical history, medication history, color blindness, and physical activity level), functional assessment of activities of daily, and general cognitive function. A trained neurologist-psychiatrist examined the potential participants to provide the final diagnosis of MCI. Individuals who met the following inclusion and exclusion criteria were invited to enroll (see Table 1). Participants were allowed or required to withdraw from the trial based on the following: development of a severe medical event during the training or follow-up period preventing continuation in the trial and reluctance from the trial.

**Table 1:** Inclusion and exclusion criteria

Inclusion criteria	Exclusion criteria
• Aged 60 years or older • Meets the diagnostic criterion of MCI [20] - report of a relative decline in cognitive functioning during the past year by the participant or informant - normal general cognitive function, including the Mini-Mental State Examination score of 25 ~ 30 Beijing version of the Montreal Cognitive Assessment score of ≤ 26 - intact activities of daily living (Instrumental Activities of Daily Living scale of Lawton and Brody <18) - absence of dementia • Have at least primary school education (≥ 5 years) • Absence of self-reported visual or auditory impairment and color blindness • Able to make an informed consent	• A history of neurological, psychiatric, and other severe medical issues that may affect brain function (i.e., epilepsy, Parkinson’s disease, head trauma, stroke, mental retardation, depression, anxiety, schizophrenia, alcohol dependency, or other addiction) • Presence of unstable cardiac disease, significant cerebrovascular disease • Taking any medications in the past 6 months may cause impaired or improved cognitive performance • Participation in other research programs related to cognitive function during this trial.

### Sample size estimation

The sample size calculation was based on primary outcome, the change in EF performance after 10 weeks. Referring to the meta-analysis of good methodological quality performed, which found that the effect size of EF training is 0.575 [0.093, 1.056] [21], the total sample size of 92 participants for this study was required at a one-sided 5% level of significance with 80% power, allowing for a 17.7% dropout rate [22].

### Randomization, allocation concealment, and blinding

After baseline assessment, eligible participants were randomly assigned to the intervention group and the wait-list control group at a ratio of 1:1 by a computer-generated random allocation sequence, resulting in 46 participants in the two groups, respectively. The random allocation sequence was sealed in opaque, sequentially numbered envelopes by a research assistant. The registered nurse and intervention coordinator were not blind to the allocated treatment, but outcome assessors and analysts were blinded to group allocation to ensure allocation concealment.

### Study interventions

#### Wait-list control intervention

Participants in the wait-list control group participated in a 40–60 min health education program on MCI twice a week over 10 weeks. The health education program was delivered by a general practitioner in the community health service center, including instruction regarding cognitive disorders, risk factors related to MCI, healthy eating, and living habits. Participants were also informed that they would receive the P-bM-tACT program after follow-up assessment. Participants were reminded via phone call to complete their education program if missing over two-week. For any questions inquired by the wait-list control group participants, general advice, but not information relating to cognitive training and brain health, was given for ethical consideration.

#### P-bM-tACT program

The P-bM-tACT program was developed by a multidisciplinary team consisting of a nursing specialist, neuropsychologist, physiotherapist, and occupational therapists following the theoretical approach of the Process Model [23], targeting EF promotion tailored to older adults with MCI. It was characterized as structured training with adaptive difficulty, consisting of eleven training tasks, with varying degrees of overlap between tasks. The training tasks are divided into three categories: a 10-min warm-up exercise, 50-min order/sequence training and expanding training using the Montessori educational wooden toys-cylinder socket blocks [24]. Among these, order/sequence training refers to the process in which study participants, without additional training obstacles, such as changes in lighting or color, rearrange a set of jumbled cylinders in a certain order based on task difficulty requirements. The specific training tasks are completed in the following order: (1) match the cylinders, (2) match the socket apertures, (3) locate the cylinders. The expanding training introduced additional obstacles, such as the use of protective eyewear or four color cards, and increased the difficulty based on the order/sequence training. The training required participants to complete tasks according to order and difficulty combined with visual-spatial ability and sensory perception. The specific sequence and tasks of training are completed in the following order: (1) recall the cylinders, (2) recall and locate cylinders, (3) recall the sequence of the colored cylinders, (4) sort the colors of the cylinders, (5) hunt for colors. In accordance with Item Response Theory (IRT) [25], the P-bM-tACT program establishes 8 distinct levels of difficulty for diverse training tasks. Thus, this program dynamically adjusts the difficulty levels of the training tasks to the participant’s current cognitive performance. Consequently, individuals who perform proficiently are advanced to a higher-level, while those who do not improve their performance remain at their current level. The protocol of the P-bM-tACT program with details has been published by our team [19].

In addition to the same health education program on MCI given to the wait-list control group, participants in the intervention group attended 3 supervised P-bM-tACT sessions/week, 60 min/session for 10 weeks in small groups with a maximum number of 6 people per group. All cognitive tasks were trained with cylinder socket blocks. There are 40 wooden cylinders and four sets of wooden socket blocks in total, 10 each per group, and each a cylinder with varying widths and depths, as same as the corresponding wooden socket block (see Supplementary File). Eight task difficulty levels were set during 10-week training (see Supplementary Table 1). Each task was presented first at the lowest difficulty level, and the subsequent training difficulty level would be raised by increasing the complexity of the task. A training deadline was set for each task using a clock to ensure that each participant could complete the training of the assigned tasks within the given time. Participants practiced this program at the community healthcare center.

Before training, we developed a detailed manual, organized into two sections, dealing first with disease knowledge and health education and secondly with training procedures. Three trained registered community nurses (one served as an instructor and the other two served as intervention facilitators) were given an intervention manual with detailed instructions and attended approximately 3 h of video conferencing training. All cognitive tasks commence with in-person instruction provided and supervised by the three trained registered community nurses following a standardized intervention protocol. Participants followed the same training schedule and were reminded via phone call to complete their training program if missing over one week by two research coordinators. Participants who failed to complete the task within the fixed time were given proper technique guides. In addition, extra training was offered for participants not finishing the cognitive training. The research coordinator would oversee the compliance of all participants at each session. All participants received bi-weekly phone calls during follow-up to balance the contact effect and reduce the loss rate.

### Outcomes and measuring instruments

#### Demographic and health characteristics

A self-designed questionnaire was used to collect demographic data and health characteristics at baseline, including age, gender, education level, residence, marital status, monthly income, occupation, living area, intellectual activity, and overall cognition function.

#### Outcome variables

The direct effect related to training gains was EF. The transfer effects related to training gains included near transfer effect (working memory) and far transfer effect (psychomotor speed).

##### Variables related to direct effects

Measurements directly relating to the training’s target cognitive domain could be more accurate and efficient in evaluating the possible cognitive effects of the intervention [26]. In this study, the direct effect related to training gains was EF, accessed by Trail Making Test A-B (containing two sub-tests: TMT-A and TMT-B) and Stroop color-word test (using software, E-Prime 2.0.10.42). The TMT-A was used to assess visual attention, and the TMT-B was used to assess task switching. The TMT A-B was timed in seconds and a faster response indicated a better cognitive performance [27]. The Stroop color-word test was used to assess the ability to inhibit cognitive interference. A higher number of correct answers in a fixed time indicated better performance [27]. Previous studies have shown that TMT A-B and Stroop color-word tests have better sensitivity and specificity for changes in EF in clinical trials of MCI [27, 28].

##### Variables related to transfer effects

The transfer effects related to training gains by the P-bM-tACT program included near transfer effects (working memory) and far transfer effects (psychomotor speed). This study measured the working memory by two subtests, including Digit span forward and backward from the Wechsler Adult Intelligence Scale-Revised [29]. Sensitivity studies of the Digit Span task have been conducted to investigate the ability to detect changes in working memory [30, 31]. A higher score in the Digit Span test indicates better performance. Previous studies with process-based training have shown relative stability and minor training effects in the far transfer effects of EF training [32]. Therefore, the psychomotor speed, as the indicator of the far transfer effects, was assessed using the auditory-visual reaction time test (model 32020 A, Lafayette instrument, US) [33] and finger tapping test (Torrance, CA 90503-5124) [28]. Previous studies have supported the reliability and validity of these tests [34, 35]. The auditory-visual reaction time test measured the time interval between the appearance of the stimulus (light, sound) and voluntary finger response [33]. The finger tapping test was used to assess psychomotor speed and it asked the participant to tap the tip of the index finger in rapid succession from a height of 15 centimeters 10 times [36].

#### Adherence rate

The following formula was used to calculate the adherence rate of the P-bM-tACT program: the number of practice programs /30 times (3 times, 10 w) ×100%.

#### Data collection

Data collection was conducted in the community healthcare center. Three trained assessors blinded to the participant group conducted a set of variable assessments. The basic characteristics were measured at baseline (T0); The outcome variables, including EF (assessed by Trail Making Test A-B: timed in seconds; Stroop color-word test: the number of correct answers in a fixed time), working memory (assessed by Digit span: the sum of the number of correctly recalled items) and psychomotor speed (assessed by Auditory-visual reaction time test: reaction time; Finger tapping test: the number of tap in a fixed time) were measured at baseline, immediately after 10-week of training (T1), and the 3-month follow-up (T2). A more detailed account of study assessments and timelines was illustrated in Supplementary Table 3.

### Statistical analysis

All statistical analyses were performed using SPSS 21.0 (SPSS Inc., USA). The method of exploratory analysis (normality test and distribution curve) was used to test the distribution state of the data. The chi-square test (categorical variables), independent-samples t-test (normally distributed variables), and Wilcoxon Rank Sum Test (not normally distributed variables) were used to examine the difference between groups on baseline demographic and outcome variables. A repeated-measures ANOVA was performed for 3-time points to evaluate the effect of group factors, time factors, and the interaction between time × group on the outcomes variables. A simple effect test was used to examine the difference among groups within each time point and between the 3-time points within each group when the interactions of time×group were significant. Effect size, one of the critical indicators to explain the potential clinical significance, was calculated using Cohen’s d [37].

## Results

**Fig. 1:**
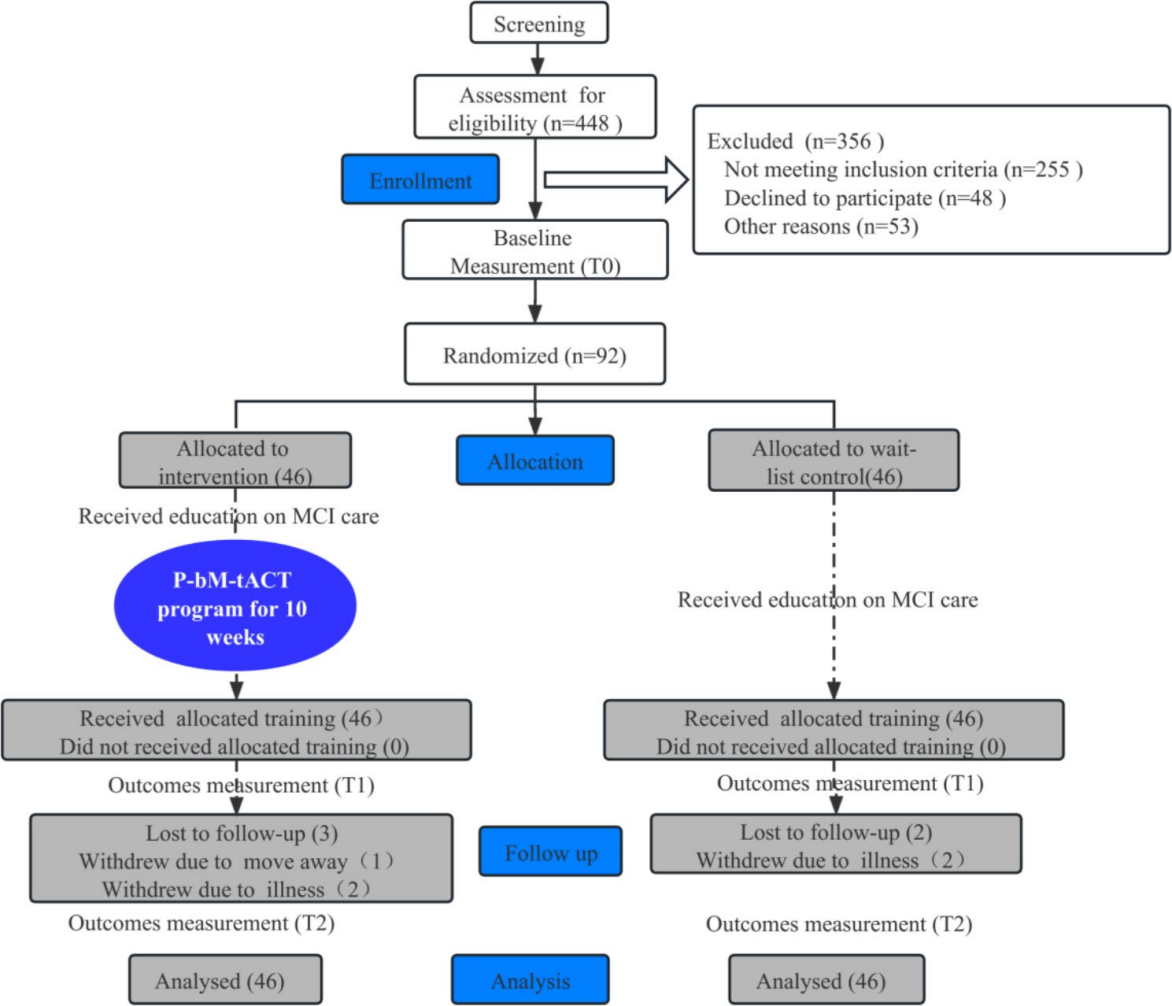
The Consolidated Standards of Reporting Trials diagram

As shown in the CONSORT flow diagram, 448 older adults in the community were screened, 92 were recruited and assigned to the intervention group or the wait-list control group at a 1:1 ratio randomly (see Fig. 1). Among them, 87 (intervention vs. control: 42 vs. 45) completed the total program and the assessment immediately after 10-week of training. 82 participants (intervention vs. control: 40 vs. 42) completed the assessment at a 3-month follow-up. There was no significant difference in the attrition rate between the two groups after a 3-month follow-up (p = 0.503), suggesting that the attrition rate did not affect study results. According to the intention-to-treat principle, missing data were imputed using the method of last observation carried forward (LOCF) [37]. Finally, data from 92 participants were analyzed. For demographic data and health characteristics of the participants and statistical comparison, please refer to Table 2 Analyses indicated no significant differences between groups in demographic characteristics, health characteristics, and baseline outcomes (all p > 0.05), suggesting that the groups had similar characteristics at baseline.

**Table 2:** Baseline data of participant demographics and health characteristics

Demographic Characteristics	Intervention Group (n = 46)	Wait-list Control Group (n = 46)	*P*
Age	74.66 ± 8.58	75.00 ± 6.59	0.830
Gender			0.527
Male	18(39.1)	21(45.7)	
Female	28(60.9)	25(54.3)	
Education level			0.836
Primary school	17(37.0)	13(28.3)	
Middle school	18(39.1)	21(45.7)	
High school	6(13.0)	6(13.0)	
Tertiary or above	5(10.9)	6(13.0)	
Residence			0.143
Living alone	8(17.4)	14(30.4)	
Living with spouse/children	38(82.6)	32(69.6)	
Marital status			0.625
Married	12(26.1)	10(21.3)	
Single (divorced, widowed)	34(73.9)	36(78.7)	
Monthly income			0.757
≤ 1000	6(13.0)	4(8.7)	
1000 ~ 2999	18(39.1)	15(32.6)	
3000 ~ 4999	14(30.5)	17(37.0)	
≥ 5000	8(17.4)	10(21.7)	
Sleeping status			0.612
Poor	11(23.9)	15(32.6)	
Fair	22(48.9)	20(43.5)	
Good	13(28.2)	11(23.9)	
Occupation			0.656
Full-time	30(65.2)	32(69.6)	
Part-time	16(34.8)	14(30.4)	
Living area			0.662
Urban	31(67.4)	29(63.0)	
Country	15(32.6)	17(37.0)	
Intellectual activity ^a^			0.807
Never	6(13.1)	8(17.4)	
Regularity ^b^	15(32.6)	13(28.3)	
Irregularity	25(54.3)	25(54.3)	
Overall cognition function			
MMSE score	21.72 ± 1.50	20.85 ± 1.38	0.327
MoCA score	19.06 ± 1.96	18.26 ± 2.20	0.063
Executive function			
Stroop Test-Word	49.89 ± 6.88	49.83 ± 6.23	0.963
Stroop Test-Color	47.77 ± 3.55	47.60 ± 3.73	0.821
Stroop Test-Color Word	17.17 ± 4.57	16.91 ± 4.70	0.790
Trail Making Test A	84.71 ± 14.53	83.59 ± 9.47	0.661
Trail Making Test B	294.43 ± 62.01	293.86 ± 64.34	0.965
Working memory			
Digit Span Forward	4.33 ± 1.19	4.30 ± 1.23	0.932
Digit Span Backward	3.50 ± 1.09	3.74 ± 0.91	0.255
Psychomotor speed			
Reaction time	2.05 ± 0.44	2.07 ± 0.43	0.887
Finger tapping test	47.61 ± 3.96	47.83 ± 3.2	0.774

**Note**: MMSE, Mini-mental State Examination; MoCA, Montreal Cognitive Assessment; a, intellectual activities were reading books, newspapers, or magazines; painting; calligraphy; playing mahjong or card games. b, regular activity defined as any intellectual activity lasting more than 1 month with a frequency of 30-min or 3-time or more per week

### Training gains of the P-bM-tACT program

#### Repeated measures ANOVAs on training effects: direct and transfer effects

A repeated-measures ANOVAs was conducted to compare the changes in all training effects at the 3-time points between the two groups. The mean values, standard deviations, and independent univariate F values were shown in Table 3. For all training effects, significant interaction effects were observed (F = 9.651 ~ 205.093, p < 0.05), as well as statistically significant main effects for group factor and time factor. A simple effects test was performed to explore the nature of the interaction by examining the difference between groups within one level of one of the independent variables.

**Table 3:** Impact of the Training Effects at Three Time-points (Group × Time) test

Outcome Measures	Time	Group (M ± SD)		ANOVA (F, P)					
		G_1_	G_2_	Group (G)		Time (T)		Group×Time	
**Direct effects**									
Trail Making Test A	T_0_	84.71 ± 14.53	83.59 ± 9.47	30.662	< 0.001	77.285	< 0.001	40.096	< 0.001
	T_1_	70.22 ± 6.38	80.66 ± 8.52						
	T_2_	62.77 ± 5.79	79.82 ± 8.07						
Trail Making Test B	T_0_	294.43 ± 62.01	293.86 ± 64.34	13.670	0.001	205.093	< 0.001	198.157	< 0.001
	T_1_	223.12 ± 60.58	290.72 ± 64.23						
	T_2_	235.39 ± 62.44	303.44 ± 65.00						
Stroop-Word	T_0_	49.89 ± 6.88	49.83 ± 6.23	11.375	0.002	99.094	< 0.001	156.332	< 0.001
	T_1_	55.96 ± 5.78	49.77 ± 6.15						
	T_2_	54.96 ± 5.69	48.74 ± 5.61						
Stroop-Color	T_0_	47.77 ± 3.55	47.60 ± 3.73	43.855	< 0.001	8.428	0.001	35.905	< 0.001
	T_1_	52.66 ± 4.42	45.79 ± 3.62						
	T_2_	51.13 ± 4.23	45.06 ± 4.57						
Stroop-Color Word	T_0_	17.17 ± 4.57	16.91 ± 4.70	9.651	0.003	12.094	< 0.001	62.177	< 0.001
	T_1_	19.43 ± 3.70	16.34 ± 4.81						
	T_2_	19.79 ± 3.50	16.04 ± 3.95						
**Near transfer effects**									
Digit Span Forward	T_0_	4.33 ± 1.19	4.30 ± 1.23	43.378	< 0.001	60.426	< 0.001	39.267	< 0.001
	T_1_	6.28 ± 1.17	5.02 ± 0.88						
	T_2_	6.52 ± 1.03	4.13 ± 0.93						
Digit Span Backward	T_0_	3.50 ± 1.09	3.74 ± 0.91	25.458	< 0.001	27.575	< 0.001	31.782	< 0.001
	T_1_	5.20 ± 1.17	3.74 ± 0.65						
	T_2_	4.35 ± 1.02	3.50 ± 0.78						
**Far transfer effects**									
Reaction Time	T_0_	2.05 ± 0.44	2.07 ± 0.43	86.991	< 0.001	12.607	< 0.001	29.115	< 0.001
	T_1_	1.55 ± 0.36	2.16 ± 0.34						
	T_2_	1.42 ± 0.18	2.30 ± 0.46						
Finger Tapping Test	T_0_	47.61 ± 3.96	47.83 ± 3.24	31.035	< 0.001	23.426	< 0.001	18.224	< 0.001
	T_1_	51.63 ± 2.58	48.43 ± 2.71						
	T_2_	52.00 ± 3.06	47.85 ± 2.74						

**Notes**: G_1_, Intervention group; G_2_, Wait-list control group; T_0_, baseline; T_1_, immediately after 10-week of training; T_2_, 3-month follow-up; ANOVA, Analysis of variance

#### Simple effect test of interaction effects on training effects: direct and transfer effects

Testing for simple main effects indicated the group factor did not have a significant effect at the baseline level on training effects (see Table 4), suggesting there were no statistical differences at baseline between the groups. Immediately after 10-week of training, the group factor had significant effects on training effects (F = 14.702 ~ 62.905, P < 0.001, Cohen’s d=-0.66 ~ 0.64), meaning intervention constructed in this study may help improve training effects. After 3-month follow-up, the group factor also had significant effects on training effects (F = 19.595 ~ 133.065, P < 0.001, Cohen’s d=-0.78 ~ 0.77), meaning training effects sustained for 3 months. Comparisons in differences separately for two groups at 3-time points were conducted. A statistically significant change was found in training effects in the intervention group immediately after 10-week of training and 3-month follow-up (F = 38.882 ~ 210.745, P < 0.001, Cohen’s d=-0.70 ~ 0.70), with significant changes in the wait-list control group for all training effects, expect for Finger Tapping Test and Digit Span Backward. Between-group effect sizes were large on training effects after 10-week of training and 3-month follow-up (Cohen’s d =-0.64 ~ 0.54) and follow-up (Cohen’s d=-0.70 ~ 0.70) compared to baseline, demonstrating improvement on training effects on after 10-week of training and 3-month follow-up were significantly better than baseline in the intervention group. Although the wait-list control group showed statistically significant differences at 3-time points, the impact on training effects differences was significantly lower than that of the intervention group.

**Table 4:** Results of Simple Effects of Interaction Effects on Training Effects

Source of Variation	G Within T_0_	G Within T_1_	G Within T_2_	T Within G_1_			T Within G_2_		
				(T_0_v.T_1_)	(T_0_v.T_2_)	(T_1_v.T_2_)	(T_0_v.T_1_)	(T_0_v.T_2_)	(T_1_v.T_2_)
**Direct effects**									
Trail Making Test A									
F	0.182	42.192**	121.797**	64.805**			15.827**		
Cohen’s d	0.05	-0.57	-0.77	0.54	0.70	0.52	0.16	0.21	0.05
Trail Making Test B									
F	0.002	31.024**	28.277**	210.745**			81.695**		
Cohen’s d	0.004	-0.48	-0.47	0.50	0.43	-0.10	0.02	-0.07	-0.10
Stroop-Word									
F	0.002	25.602**	28.213**	173.187**			14.168**		
Cohen’s d	0.001	0.46	0.48	-0.43	-0.37	0.09	0.004	0.09	0.09
Stroop-Color									
F	0.055	62.905**	47.353**	39.361**			9.533**		
Cohen’s d	0.02	0.64	0.56	-0.52	-0.39	0.17	0.24	0.29	0.09
Stroop-Color Word									
F	0.097	14.702**	26.712**	67.080**			5.157*		
Cohen’s d	0.03	0.34	0.45	-0.26	-0.31	-0.05	0.06	0.10	0.03
**Near transfer effects**									
Digit Span Forward									
F	0.006	29.156**	133.065**	74.004**			15.541**		
Cohen’s d	0.01	0.52	0.77	-0.64	-0.70	-0.11	-0.32	0.08	0.44
Digit Span Backward									
F	2.028	56.727**	19.595**	38.882**			2.932		
Cohen’s d	-0.12	0.61	-0.42	-0.60	-0.37	0.36	0.001	0.14	0.16
**Far transfer effects**									
Reaction Time									
F	0.017	61.246**	122.220**	47.603**			4.154*		
Cohen’s d	-0.02	-0.66	-0.78	0.53	0.68	0.22	-0.12	-0.25	-0.17
Finger Tapping Test									
F	0.080	41.675**	58.897**	59.774**			0.624		
Cohen’s d	-0.03	0.52	0.58	-0.52	-0.53	-0.07	-0.10	-0.03	0.11

Notes: G WITHIN T_0_: The simple effect of G at T_0_ level (the comparative results on levels of baseline in intervention and wait-list control group); G WITHIN T_1_: the comparative results of the two groups on immediately after 10-week of training; G WITHIN T_2_: the comparative results of the two groups after 3-month follow-up; T WITHIN G_1_: the comparative results of the intervention group at baseline, after 10-week of training and 3-month follow-up; T WITHIN G_2_: the comparative results of the wait-list control group at baseline, after 10-week of training and 3-month follow-up; *P < 0.05; **P < 0.01; *P < 0.05; **P < 0.01

## Discussion

### Summary of the findings

The presented study assessed the efficacy of the P-bM-tACT program targeting EF promotion among older adults with MCI and further examined the transfer and sustainability of effects over a three-month observation period. The study’s findings revealed that the P-bM-tACT program provided immediate benefits for the training effects, with direct effects and transfer effects observed in work memory and psychomotor speed. Furthermore, the gains of the program were sustained for 3 months, and no training-related adverse events occurred throughout the study. The intervention group recorded an 83.4% adherence rate, indicating that the P-bM-tACT program is highly feasible and acceptable for community-dwelling older adults with MCI.

### Comparison with other studies

#### Direct effects of the P-bM-tACT program targeting EF promotion

The study’s findings indicate that the P-bM-tACT program was effective in improving EF performance when compared to the wait-list control group. Furthermore, similar gains were observed at 3-month follow-up. These results are consistent with previous studies, which demonstrate the modifiability of EF through cognitive training [6, 7, 32]. The cognitive performance that older adults achieve and retain after undergoing process-based cognitive training might be attributed to their cognitive plasticity. Cognitive plasticity in older adults is preserved to a large extent, and even fairly short cognitive training programs can produce (partially specific) training and transfer effects [38]. Distinct design characteristics of cognitive training program may explain those improvements. The efficacy or applicability of training mainly depends on the validity and targeted training content or tasks [39]. The P-bM-tACT program is specifically designed to enhance EF, a cognitive function that holds a vital position in cognitive architecture, particularly in cognitive executive control processes. One of the program’s training tasks, such as matching the socket apertures, is geared towards improving working memory and mental flexibility, both of which are sub-components of EF. By participating in this training, participants in the intervention group achieved significant improvements in their test scores as compared to the baseline. Moreover, cognitive gains were associated with the dose-response to the training sessions, as suggested by two meta-analysis studies [21, 40]. These studies revealed that the average duration of cognitive training was 27.57 h (SD = 24.60), and the training effects were significant with a minimum of 3 sessions per week. Thus, the training sessions of the P-bM-tACT program for older adults with MCI in this study were set to 3-time per week (60 min/times) for 10 weeks (30 total training sessions). It is anticipated that providing sufficient training time would lead to further improvements in the EF performance for participants. Furthermore, training gains are more reliable and accurate when selecting evaluation indicators directly and specifically associated with the targeted cognitive domains [39]. Neuropsychological tests, such as TMT A-B and Stroop color-word interference test, used in this study, are commonly employed to estimate EF [27, 28]. These tests are considered to be more sensitive to changes in EF and, therefore, may provide a better reflection of the actual training gains.

#### Transfer effects of the P-bM-tACT program

The present study aimed to comprehensively examine whether the P-bM-tACT program can produce both a near transfer effect (i.e., working memory) and a far transfer effect (i.e., psychomotor speed). One predominant hypothesis assumes that transfer effects are likely to occur if the trained and untrained cognitive domains share a common neural network or rely on similar neural activations [41], it is expected that transfer effects of the P-bM-tACT program are possible. In other words, transfer effects from the trained to the untrained cognitive domains can be expected when two tasks share common components, such as working memory and mental flexibility, which are subcomponents of EF, both in terms of cognitive processing steps and reliance on similar neural activation [42]. This also reflects the potential mediating mechanisms that direct effects may play an important role in the generation of transfer effects.

#### Training gains of the near transfer effect

In this study, the near transfer effect on working memory was observed after the intervention and was found to be sustained over 3-month follow-up period. It is well-established that performance on working memory, as assessed by the digit span test, is associated with executive control or brain regions where EF dominates, such as the left frontal lobe and posterior parietal white matter [43–45]. Previous studies have also shown that the digit span test depends on the central-executive performance component of working memory [46, 47]. This component was effectively enhanced by the P-bM-tACT program, thereby facilitating the transfer effect of working memory improvement. These findings are consistent with the theory of Dahlin, which suggests that transfer effects are likely to occur when training and transfer tasks engage overlapping processing components [48]. In addition, adaptive training is an essential feature for effective training tasks [49], as it promotes more special training effects, such as perceptual speed, attention, and mental rotation, as well as transfer effects than non-adaptive training [18]. The P-bM-tACT program emphasizes training adaptation, which means that the task difficulty is continuously adjusted to the participant’s cognitive performance level during training to enhance training and potential transfer effects. Researchers have suggested that adaptive training may improve EF and working memory by inducing the release of dopamine-induced neurotrophic factors in the dorsolateral frontostriatal loop, which involves interaction between the prefrontal cortex and caudate nucleus [50]. Therefore, the improvement in the working memory observed in participants of this study may be a transfer effect produced by the P-bM-tACT program.

#### Training gains of the far transfer effect

The results of this study indicated that after 10-week of training intervention, the far transfer effect in psychomotor speed was observed and sustained over 3-month follow-up period. Previous studies have mostly utilized reaction time and finger tapping tests to evaluate psychomotor speed, attention control, and cognitive flexibility [51, 52]. In the reaction time test, participants were required to press the corresponding color buttons quickly and accurately according to instructions given by three trained assessors while ignoring or countering the color cues presented by the device voice. These abilities mentioned above were more dependent on advanced cognitive functions, such as EF and processing ability, which were the focus of the intensive training in this study. Therefore, transfer effects were more likely to have occurred. Age-related cognitive decline is associated with cortical disconnection, axonal degeneration, and myelin degeneration which impair cognitive functions such as attention, working memory, psychomotor speed and EF [53–55]. The study by Wu et al. [56] showed that cognitive training targeting EF can improve psychomotor speed in healthy older adults by increasing the functional connectivity between the anterior cingulate cortex (an executive control region) and the posterior parietal cortex. It is possible that the P-bM-tACT program may produce a transfer effect on psychomotor speed through similar underlying mechanisms. Several imaging studies have shown that executive-related cognitive functions are mainly dominated by the prefrontal and parietal cortex, while tasks involving outcome feedback are mainly dominated by the posterior cortical regions, such as the hippocampus, parahippocampal gyrus, and entorhinal cortex [55, 57]. The posterior cortical regions are indirectly influenced by executive control regions [58], which may promote increased psychomotor speed, and this might be a potential mechanism to explain the far transfer effect produced by the P-bM-tACT program. However, the results of this study still require further verification by objective measures.

#### Sustainability of the training gain

The persistent effect of training, one of the most valuable cognitive training features, has been found to be an important precipitating factor in reducing the risk of progressive dementia due to MCI [59]. This study demonstrated that the P-bM-tACT program has a direct effect on EF and transfer effects on other cognitive functions, which were sustained for up to 3-month follow-up. Previous studies of cognitive training targeting specific cognitive domains in individuals with MCI have shown similar results [58, 59]. Klimova has highlighted the importance of sustaining training-induced cognitive benefits during the follow-up phase to ensure the sustainability of the cognitive training program in healthcare centers or home settings [60]. In this study, participants in the intervention group were given the P-bM-tACT program intervention manual and encouraged to apply what they learned in training to their daily activities. However, previous studies on the process-based cognitive training have rarely tracked the cognitive benefits of MCI participants over time, resulting in a lack of consensus on the sustainability of the cognitive training-induced cognitive benefit across this population. Therefore, the potential long-term benefits should be confirmed in high-quality longitudinal studies in future research.

#### Study strengths and implications

Two strong points need to be highlighted in this study. Firstly, we demonstrated the effectiveness, feasibility, and acceptability of a nurse-led cognitive training program for older adults with MCI in the community setting. In China, where community healthcare resources are limited, access to professional cognitive and mental health therapists is only available in a few large cities [61]. Therefore, this study developed a nurse-led cognitive training program based on the process model. The design of the P-bM-tACT program was easy to complete and ensured compliance motivation of older adults with MCI. Secondly, the focus of this study was to explore the direct and transfer effects of the P-bM-tACT program. The results demonstrated that the improvement of EF during the intervention facilitated changes in participants’ working memory and processing speed simultaneously. Additionally, a 3-month follow-up observation was conducted to confirm whether the observed direct effects and transfer effects of training were sustained over time. Assessing transfer and sustained effects could enhance the precision and comprehensive implementation of cognitive training, tailored to individuals’ diverse needs. Understanding that different types of training should contain transfer effects highlights the potential value of optimally matched cognitive training approaches to achieve cognitive benefits.

This study offers important implications for nursing practice in community settings. The nurse-led health management P-bM-tACT program, developed in this study is an encouraging step towards involving community nurses in the cognitive health of older adults with MCI. Community nurses can provide cognitive education and self-management activities to promote cognitive health in community-dwelling older adults with MCI. Therefore, specialized cognitive training skills for community nurses are essential to successfully implement cognitive training programs. Second, given the direct and transfer effects on cognitive benefits of the P-bM-tACT program, nurses at the primary health services center should encourage older adults with MCI to engage in periodic cognitive training. Actions should be taken to enhance adherence to cognitive training among older adults with MCI, such as dynamic adjustment of difficulty of the training task, peer or family support, and schedule development. These healthy behaviors will reinforce and prolong the beneficial effect of training in older adults with MCI.

#### Limitations and future research directions

Several limitations of this study need to be mentioned. The sample population in this study may not fully represent the overall characteristics of older adults with MCI due to the exclusion of those with color blindness and education levels below the primary school. Future studies will be critical to confirm the applicability of these benefits to diverse populations. Besides, we employed a wait-list control group instead of an active control group, as a previous study showed that the effect size might be similar between active control and passive control [62]. The debate about the treatment effect difference between active and passive control (no intervention or regular health education) groups is ongoing. Nevertheless, an active control group, such as different cognitive training strategies or non-adaptive cognitive training, should be added to future studies to reduce this intervention bias possibly.

## Conclusions

The growing interest in cognitive interventions for MCI promoted the need to increase its application to prevent or slow further cognitive decline. The presented study confirmed that the P-bM-tACT program enabled older adults with MCI to improve and sustain training effects (direct and transfer effect) and demonstrated the sustainability of training-induced effects over time. Future research with medical imageology data is proposed to identify the mechanisms through which transfer effects produce and to refine the understanding of the transferability scope and limits.

## Electronic supplementary material

Below is the link to the electronic supplementary material.

Supplementary Material 1? Instruments for the P-bM-tACT program.

Supplementary Material 2? Supplement Table 1-Training tasks of the P-bM-tACT program.

Supplementary Material 3? Supplement Table 2-Summary of study assessments and timelines.

## Data Availability

The datasets used and/or analyzed during the current study are available from the corresponding author on reasonable request.

## List of Abbreviations

MCI: Mild cognitive impairment
EF: Executive function
P-bM-tACT: Process-based multi-task adaptive cognitive training
MoCA: Montreal Cognitive Assessment
MMSE: Mini-mental State Examination

## References

[1] Liang JH, Shen WT, Li JY (2019). The optimal treatment for improving cognitive function in elder people with mild cognitive impairment incorporating bayesian network meta-analysis and systematic review. Ageing Res Rev.

[2] Chehrehnegar N, Shati M, Esmaeili M (2022). Executive function deficits in mild cognitive impairment: evidence from saccade tasks. Aging Ment Health.

[3] Diamond A (2013). Executive functions. Annu Rev Psychol.

[4] Chang YL, Jacobson MW, Fennema-Notestine C (2010). Level of executive function influences verbal memory in amnestic mild cognitive impairment and predicts prefrontal and posterior cingulate thickness. Cereb Cortex.

[5] Rosenberg A, Solomon A, Jelic V (2019). Progression to dementia in memory clinic patients with mild cognitive impairment and normal β-amyloid. Alzheimers Res Ther.

[6] Zhong D, Chen L, Feng Y (2021). Effects of virtual reality cognitive training in individuals with mild cognitive impairment: a systematic review and meta-analysis. Int J Geriatr Psychiatry.

[7] Yu D, Li X, Lai FH. The effect of virtual reality on executive function in older adults with mild cognitive impairment: a systematic review and meta-analysis. Aging Ment Health. 2022;1–11. 10.1080/13607863.2022.2076202. 10.1080/13607863.2022.2076202 35635486

[8] Audiffren M, André N, Baumeister RF (2022). Training willpower: reducing costs and valuing effort. Front Neurosci.

[9] Shin M, Lee A, Cho AY (2020). Effects of process-based cognitive training on memory in the healthy Elderly and patients with mild cognitive impairment: a Randomized Controlled Trial. Psychiatry Investig.

[10] Gavelin HM, Boraxbekk CJ, Stenlund T (2015). Effects of a process-based cognitive training intervention for patients with stress-related exhaustion. Stress.

[11] Studer-Luethi B, Boesch V, Lusti S, et al. Fostering cognitive performance in older adults with a process- and a strategy-based cognitive training. Neuropsychol Dev Cogn B Aging Neuropsychol Cogn. 2022;1–23. 10.1080/13825585.2022.2105298. 10.1080/13825585.2022.2105298 35912438

[12] Owen AM, Hampshire A, Grahn JA (2010). Putting brain training to the test. Nature.

[13] Simons DJ, Boot WR, Charness N (2016). Do “Brain-Training” Programs Work?. Psychol Sci Public Interest.

[14] Barnett SM, Ceci SJ (2002). When and where do we apply what we learn? A taxonomy for far transfer. Psychol Bull.

[15] Anguera JA, Boccanfuso J, Rintoul JL (2013). Video game training enhances cognitive control in older adults. Nature.

[16] Rebok GW, Ball K, Guey LT (2014). Ten-year effects of the advanced cognitive training for independent and vital elderly cognitive training trial on cognition and everyday functioning in older adults. J Am Geriatr Soc.

[17] Hill NT, Mowszowski L, Naismith SL, Chadwick VL, Valenzuela M, Lampit A (2017). Computerized cognitive training in older adults with mild cognitive impairment or dementia: a systematic review and Meta-analysis. Am J Psychiatry.

[18] Kardys C, Küper K, Getzmann S (2022). A comparison of the Effects of short-term physical and combined multi-modal training on cognitive functions. Int J Environ Res Public Health.

[19] Zhao X, Wang L, Ge C, Liu X, Chen M, Zhang C (2020). Effect of process-based Multi-Task Cognitive Training Program on executive function in older adults with mild cognitive impairment: study rationale and Protocol Design for a Randomized Controlled Trial. Front Psychiatry.

[20] Petersen RC, Morris JC (2005). Mild cognitive impairment as a clinical entity and treatment target. Arch Neurol.

[21] Sherman DS, Mauser J, Nuno M (2017). The efficacy of cognitive intervention in mild cognitive impairment (MCI): a Meta-analysis of outcomes on neuropsychological measures. Neuropsychol Rev.

[22] Chandler MJ, Parks AC, Marsiske M (2016). Everyday impact of cognitive interventions in mild cognitive impairment: a systematic review and Meta-analysis. Neuropsychol Rev.

[23] Kliegel M, Altgassen M, Hering A (2011). A process-model based approach to prospective memory impairment in Parkinson’s disease. Neuropsychologia.

[24] Brandão DF, Martín JI (2012). Montessori method applied to dementia-literature review. Rev Gaucha Enferm.

[25] Reise SP, Rodriguez A (2016). Item response theory and the measurement of psychiatric constructs: some empirical and conceptual issues and challenges. Psychol Med.

[26] Kinsella GJ, Mullaly E, Rand E (2009). Early intervention for mild cognitive impairment: a randomised controlled trial. J Neurol Neurosurg Psychiatry.

[27] Periáñez JA, Lubrini G, García-Gutiérrez A (2021). Construct validity of the Stroop Color-Word Test: influence of speed of visual search, Verbal Fluency, Working Memory, Cognitive Flexibility, and conflict monitoring. Arch Clin Neuropsychol.

[28] Godefroy O, Martinaud O, Narme P (2018). Dysexecutive disorders and their diagnosis: a position paper. Cortex.

[29] Ruchinskas R (2019). Wechsler adult intelligence scale-4th edition digit span performance in subjective cognitive complaints, amnestic mild cognitive impairment, and probable dementia of the Alzheimer type. Clin Neuropsychol.

[30] Conway AR, Kane MJ, Bunting MF, Hambrick DZ, Wilhelm O, Engle RW (2005). Working memory span tasks: a methodological review and user’s guide. Psychon Bull Rev.

[31] Gray NS, Hurdman J, Munafò MR (2003). Inhibition, working memory and cigarette smoking. Psychopharmacology.

[32] Sandberg P, Stigsdotter Neely A (2016). Long-term effects of executive process training in young and old adults. Neuropsychol Rehabil.

[33] Suarez S, Eynard B, Granon S (2021). A dissociation of attention, executive function and reaction to Difficulty: development of the MindPulse Test, a Novel Digital Neuropsychological Test for Precise quantification of perceptual-motor decision-making processes. Front Neurosci.

[34] Delis DC, Kramer JH, Kaplan E, et al. California Verbal Learning Test®–Second Edition (CVLT®–II) manual. The Psychological Corporation; 2003.

[35] Wang M, Gao L, Gao J (2018). Age-related changes in the performance of the auditory-visual reaction time task in chinese adults. Front Aging Neurosci.

[36] Gouttebarge V, Andersen TE, Cowie C (2019). Monitoring the health of transitioning professional footballers: protocol of an observational prospective cohort study. BMJ Open Sport Exerc Med.

[37] Larner AJ (2014). Effect size (Cohen’s d) of cognitive Screening Instruments examined in pragmatic diagnostic accuracy studies. Dement Geriatr Cogn Dis Extra.

[38] Bherer L (2015). Cognitive plasticity in older adults: effects of cognitive training and physical exercise. Ann N Y Acad Sci.

[39] Louisa GS, Alexandra KG, Jonathan PS (2016). A randomized, placebo-controlled proof-of-concept trial of adjunctive topiramate for alcohol use disorders in bipolar disorder. Am J Addict.

[40] Robert P, Manera V, Derreumaux A (2020). Efficacy of a web app for cognitive training (MeMo) regarding cognitive and behavioral performance in people with Neurocognitive Disorders: Randomized Controlled Trial. J Med Internet Res.

[41] Kelly ME, Loughrey D, Lawlor BA (2014). The impact of cognitive training and mental stimulation on cognitive and everyday functioning of healthy older adults: a systematic review and meta-analysis. Ageing Res Rev.

[42] Thompson TW, Waskom ML, Garel KL (2013). Failure of working memory training to enhance cognition or intelligence. PLoS ONE.

[43] Nixon SJ, Lewis B (2019). Cognitive training as a component of treatment of alcohol use disorder: a review. Neuropsychology.

[44] Albinet CT, Boucard G, Bouquet CA (2012). Processing speed and executive functions in cognitive aging: how to disentangle their mutual relationship?. Brain Cogn.

[45] Hirsiger S, Koppelmans V, Mérillat S (2017). Executive functions in healthy older adults are differentially related to macro- and Microstructural White Matter characteristics of the cerebral lobes. Front Aging Neurosci.

[46] Emrani S, Libon DJ, Lamar M (2018). Assessing Working memory in mild cognitive impairment with serial Order Recall. J Alzheimers Dis.

[47] Zheng Z, Lang M, Wang W (2019). False recognition of emotionally categorized pictures in Young and older adults. Front Psychol.

[48] Ichihara-Takeda S, Takeda K, Ikeda N et al. Neuropsychological Assessment of a New Computerized Cognitive Task that Was Developed to Train Several Cognitive Functions Simultaneously. Front Psychol. 2016;7:497. 10.3389/fpsyg. 2016.00497. 10.3389/fpsyg.2016.00497 PMC4828453 27148110

[49] Dahlin E, Neely AS, Larsson A (2008). Transfer of learning after updating training mediated by the striatum. Science.

[50] Moustafa AA, Poletti M (2013). Neural and behavioral substrates of subtypes of Parkinson’s disease. Front Syst Neurosci.

[51] Gale CR, Harris A, Deary IJ (2016). Reaction time and onset of psychological distress: the UK Health and Lifestyle Survey. J Epidemiol Community Health.

[52] Ashendorf L, Vanderslice-Barr JL, McCaffrey RJ (2009). Motor tests and cognition in healthy older adults. Appl Neuropsychol.

[53] Hedden T, Gabrieli JD (2004). Insights into the ageing mind: a view from cognitive neuroscience. Nat Rev Neurosci.

[54] Raz N, Kennedy KM, Jagust W, D’Esposito M (2009). A systems approach to the aging brain: neuroanatomic changes, their modifiers, and cognitive correlates. Imaging the aging brain.

[55] Knopman DS, Griswold ME, Lirette ST (2015). Vascular imaging abnormalities and cognition: mediation by cortical volume in nondemented individuals: athero-sclerosis risk in communities-neurocognitive study. Stroke.

[56] Wu L, Zhang H, Qi R et al. Long-term cognitive improvement after cognitive training for the elderly: a 6-month follow-up functional MRI study. Neuropsych -ology, Development, and Cognition. Section B, Aging, Neuropsychology and Cognition.2017; 24(2):156–173.

[57] Brugulat-Serrat A, Salvadó G, Operto G (2020). White matter hyperintensities mediate gray matter volume and processing speed relationship in cognitively unimpaired participants. Hum Brain Mapp.

[58] Lemke NC, Werner C, Wiloth S (2019). Transferability and sustainability of Motor-Cognitive Dual-Task Training in patients with dementia: a Randomized Controlled Trial. Gerontology.

[59] Weng W, Liang J, Xue J (2019). The transfer Effects of Cognitive Training on Working Memory among Chinese older adults with mild cognitive impairment: a Randomized Controlled Trial. Front Aging Neurosci.

[60] Klimova B (2016). Computer-based cognitive training in aging. Front Aging Neurosci.

[61] Zhang H, Wang J, Sun T (2018). A randomized controlled trial of combined executive function and memory training on the cognitive and noncognitive function of individuals with mild cognitive impairment: study rationale and protocol design. Alzheimers Dement (N Y).

[62] Lawlor-Savage L, Goghari VM, Dual N-Back (2016). Working memory training in healthy adults: a Randomized comparison to Processing Speed Training. PLoS ONE.

