# Supplementary material for: Transferability and sustainability of process-based multi-task adaptive cognitive training in community-dwelling older adults with mild cognitive impairment: a randomized controlled trial
Source: BMC Psychiatry. 2023 Jun 12;23:418. doi: 10.1186/s12888-023-04917-3 (PMC10259063; doi:10.1186/s12888-023-04917-3)

**Supplement Figure**

**Cylinder socket blocks**

There are 40 wooden cylinders and four sets of wooden socket blocks in total, 10 each per group and each a cylinder with varying widths and depths as same as the corresponding wooden socket block. The first set of cylinders increases in depths and widths, the second set of cylinders only increases in depths with the same widths, the third set of cylinders only increases in widths with the same depths, and the last set of cylinders increases in widths but decreases in depths.


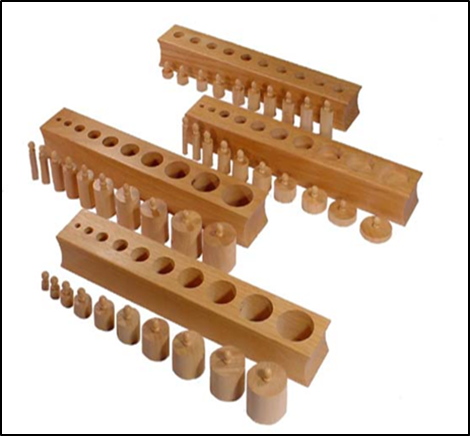


A

B

C

D

**Fig. 1 Instruments for the P-bM-tACT program**

| Cylinder number | Width | Depth |
| --- | --- | --- |
| A | increase | increase |
| B | same | increase |
| C | increase | same |
| D | increase | decrease |

Cylinder structure

Cylinder socket arrangement and combination

| **Compound mode** | **Concrete form** |
| --- | --- |
| **one group** | B、C、D、A |
| **two groups** | B+C、B+D、C+D、C+A |
| **three groups** | B+C+D、C+D+A、A+B+C、A+B+D |
| **for groups** | A+B+C+D |

In different levels of tasks, participants were asked to start with a group of cylinders socket blocks first (participants choose one from ABC and D). After completing a group of cylinder socket blocks, participants were asked to start with two groups of cylinder socket blocks (for example, participants choose B+D). After completing two groups of cylinder socket blocks, participants were asked to start with three groups (for example, participants choose A+B+C). By that analogy, participants complete tasks.


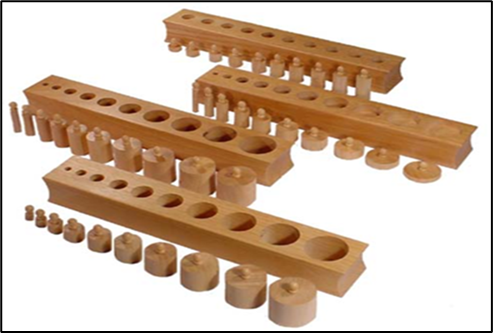

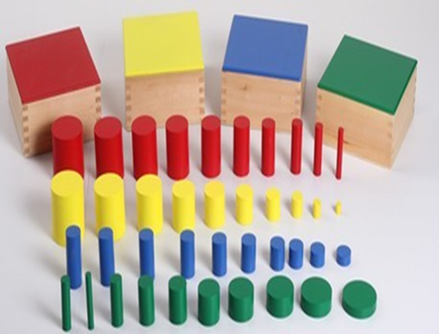


Cylinder socket blocks Colored cylinder socket blocks


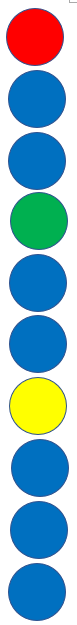

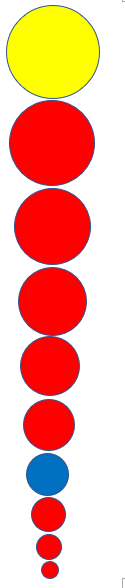

Supplement: Supplementary file 1 — Supplementary Material 1? Instruments for the P-bM-tACT program. [file 12888_2023_4917_MOESM1_ESM.docx]
