# Supplementary material for: Transferability and sustainability of process-based multi-task adaptive cognitive training in community-dwelling older adults with mild cognitive impairment: a randomized controlled trial
Source: BMC Psychiatry. 2023 Jun 12;23:418. doi: 10.1186/s12888-023-04917-3 (PMC10259063; doi:10.1186/s12888-023-04917-3)

**Supplement Table 1**

**Supplement Table 1 Training tasks of the P-bM-tACT program**

| Categories | Tasks | Targeted  domain | Difficulty  levels/rounds | number of hours | A brief description of the tasks |
| --- | --- | --- | --- | --- | --- |
| Warm-up exercise | Recall the names | Memory | 0/20 | 10min | Firstly, introduce yourself one by one (i.e., name, age, occupation). Then, inform participants to remember these names. The participants are asked to write down the names of the other five on white paper according to their memory at the end of cognitive training. |
|  | You act I guess | Working memory | 0/1 |  | Firstly, participants were divided into three groups of two. Then, one participant facing the audience uses the most accurate body language to express the meaning of words, while other partici  -pants with their backs to the audience are asked to guess those words. Each group is limited to 2 minutes. |
| Order/sequence training | Match the cylinders | Executive function | 1/64 | 50min | Firstly, take any set of socket cylinder socket, make the cylinder out of the socket, placed near the corresponding socket block, then throw the order of a group of cylinders into confusion. The participants will be required to place each cylinder into appropriate holes in a long wooden socket block with the left-to-right or the right-to-left order. There are sixty-four cylinder socket combinations. With more socket cylinders and change in combination model shown, the difficulty level increases. |
|  | Match the socket apertures |  | 2/64 |  | Firstly, take any set of socket cylinder socket, make the cylinder out of the socket, placed near the corresponding socket block, then throw the order of a group of cylinders into confusion. A wooden cylinder was selected and placed it into the participant's hand. The participants will be required to find the corresponding position of each cylinder and place it into appropriate holes in a long wooden socket block. With more socket cylinders and changes in the combination model shown, the difficulty level increases. |
|  | Locate the cylinders |  | 3/64 |  | Firstly, take any set of socket cylinder socket on the table, make the cylinder out of the socket, placed socket block on another table. The participants will be required to select suitable cylinders that were shown in the first group and place each cylinder into appropriate holes in a long wooden socket block with the left-to-right or the right-to-left order according to their memory. With more socket cylinders and changes in the combination model shown, the difficulty level increases. |
| Expanding training | Recall the cylinders |  | 4/64 |  | The participants will be required to complete a task on level 1 (Match the cylinders) with eyes closed. With more socket cylinders and changes in the combination model shown, the difficulty level increases. |
|  | Recall and locate the cylinders |  | 5/64 |  | The participants will be required to complete a task on level 2 (Match the socket apertures) with eyes closed. With more socket cylinders and changes in the combination model shown, the difficulty level increases. |
|  | Recall the sequence of the colored cylinders |  | 6/64 |  | Firstly, take any set of socket cylinder socket with colors * (Brand Name: DAWONMONTESSORI; Model Number: DW 1002 ). Then the participants will be required to complete a task on level 1 (Match the cylinders). With more socket cylinders and changes in the combination model shown, the difficulty level increases. |
|  | Sort the colors of the cylinders |  | 7/4 |  | Firstly, color wooden cylinders* with characters on their surface are shown. The color of characters includes red, yellow, blue and green, orange, pink, purple and black. The characters include red, orange, yellow, green, blue, pink, purple, black, white and gray. Then the participants will be required to describe the color of the words on the surface of the cylinder and complete the categorization and sorting. |
|  | Hunt for the colors |  | 8/10 |  | Firstly, take any set of socket cylinder socket with colors (red or green, blue, yellow) are shown. Then the participants will be required to complete a task on level 1 (Match the cylinders) according to cylinder color card (the time of observing card should not exceed 60 seconds). With more socket cylinders and changes in the combination model shown, the difficulty level increases. |

* The Cylinders are four sets of wooden cylinders, each a different color with varying widths and depths as same as the cylinder blocks.

Detail description

1. Match the socket apertures


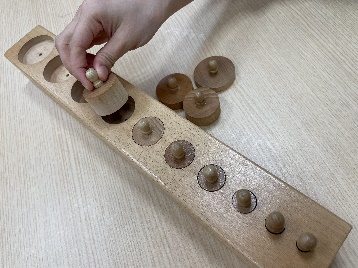

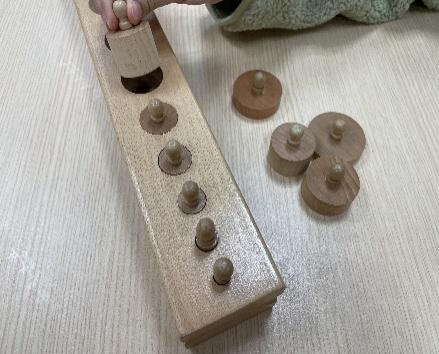

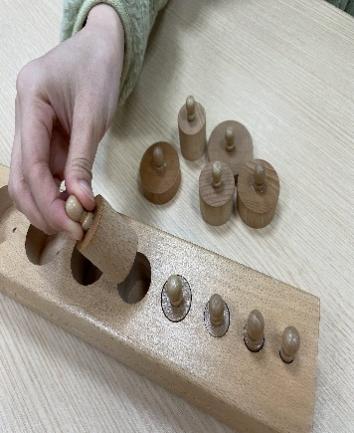

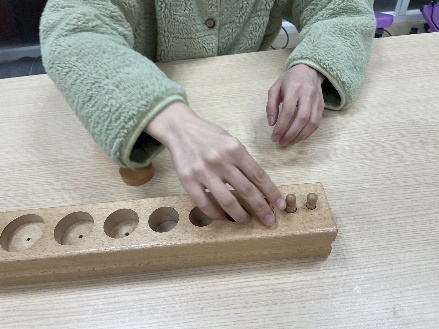

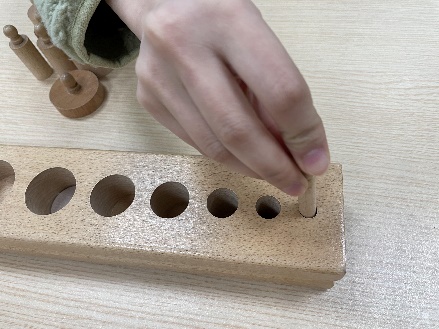


1. Match the socket apertures


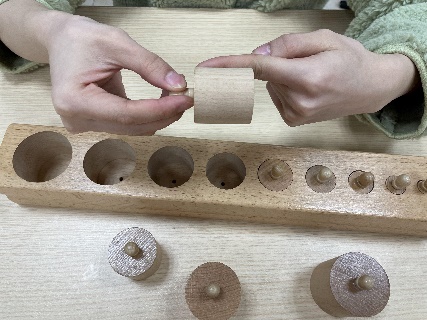

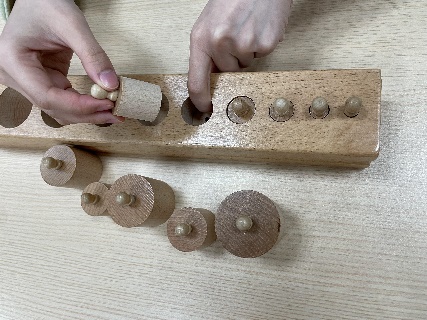


1. Locate the cylinders


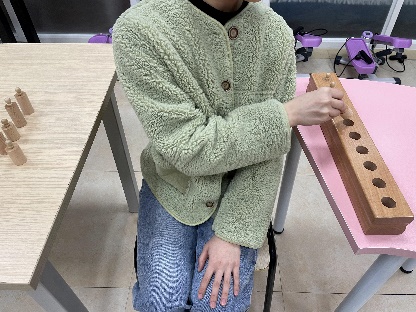

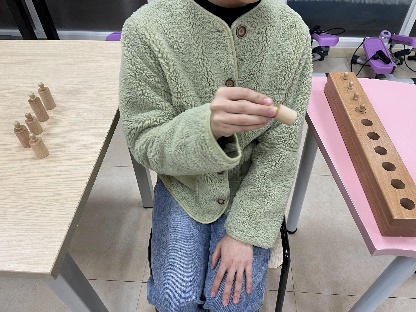

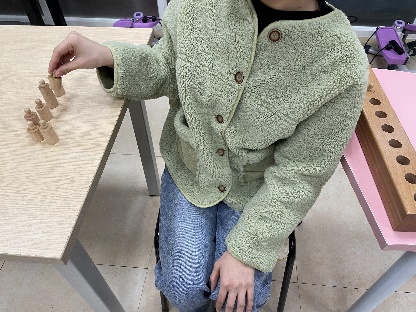


1. Recall and locate the cylinders


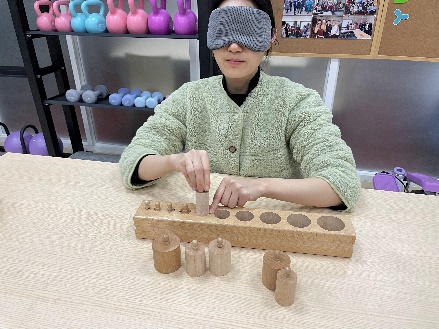

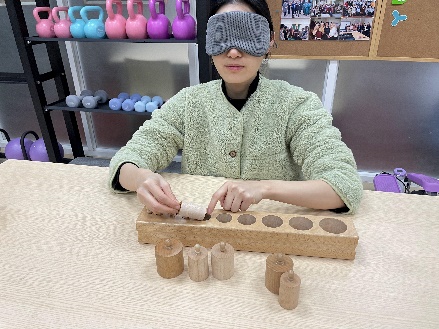

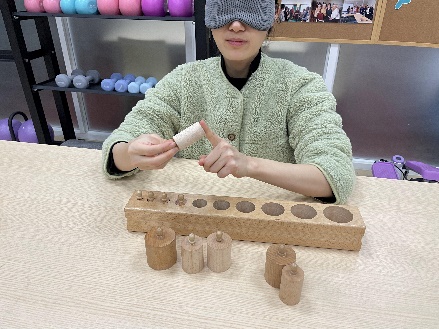

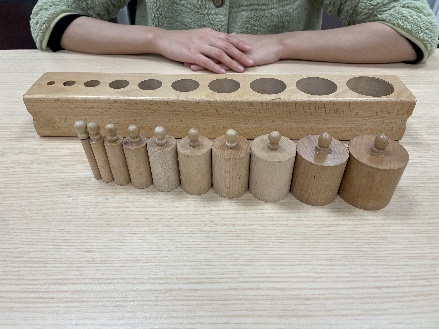


1. Recall and locate the cylinders


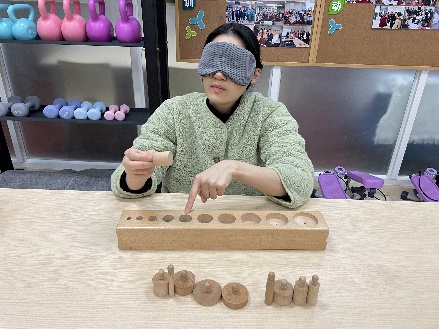

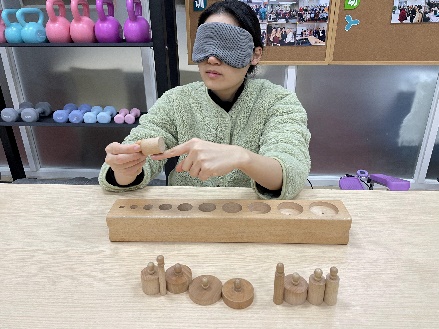

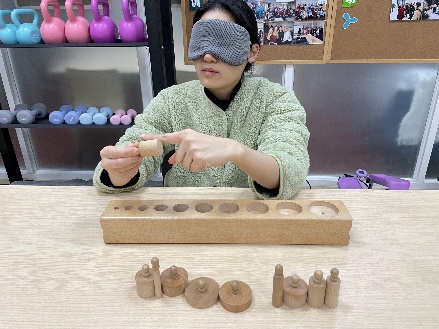

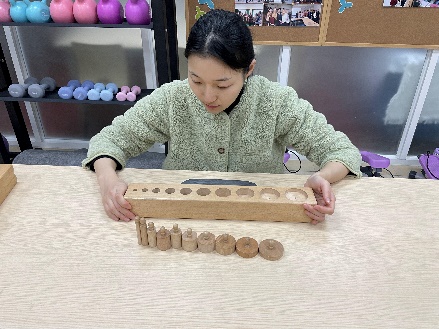


1. Recall the sequence of the colored cylinders


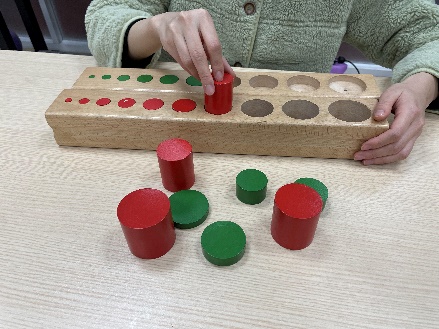

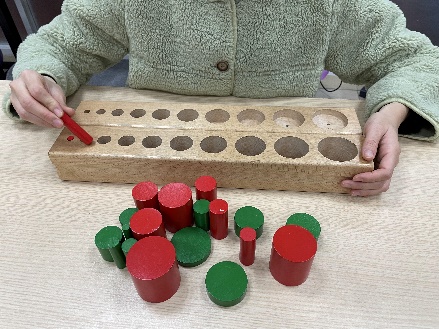

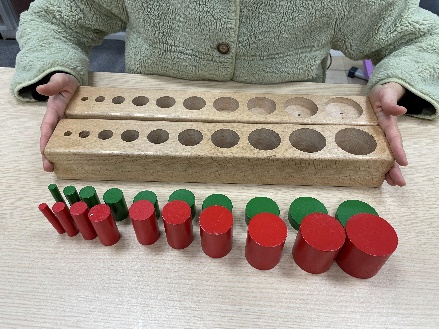


1. Sort the colors of the cylinders


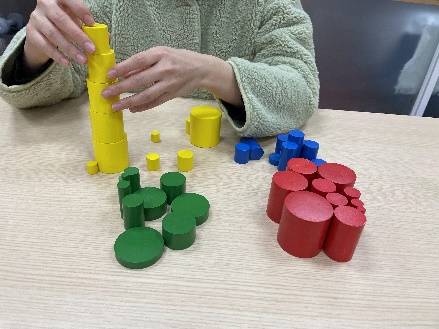

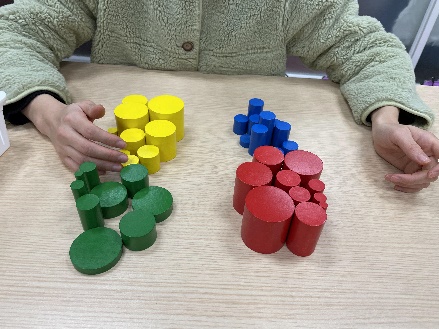


1. Hunt for the colors


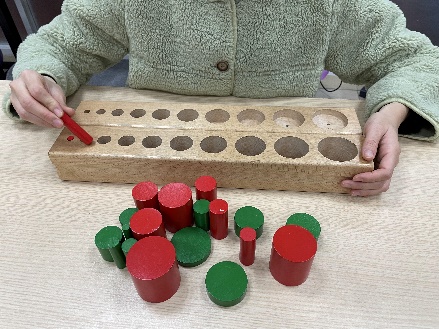

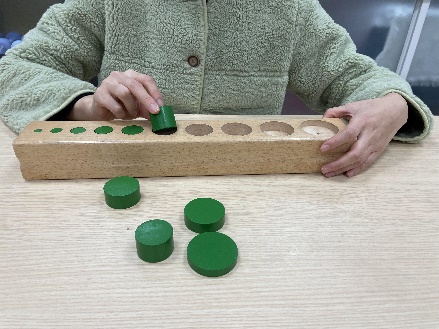


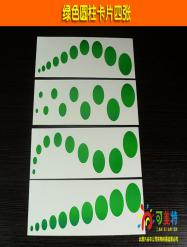

Supplement: Supplementary file 2 — Supplementary Material 2? Supplement Table 1-Training tasks of the P-bM-tACT program. [file 12888_2023_4917_MOESM2_ESM.docx]
