# Supplementary material for: Transferability and sustainability of process-based multi-task adaptive cognitive training in community-dwelling older adults with mild cognitive impairment: a randomized controlled trial
Source: BMC Psychiatry. 2023 Jun 12;23:418. doi: 10.1186/s12888-023-04917-3 (PMC10259063; doi:10.1186/s12888-023-04917-3)
Supplement: Supplementary file 3 — Supplementary Material 3? Supplement Table 2-Summary of study assessments and timelines. [file 12888_2023_4917_MOESM3_ESM.docx]

**Supplement Table 2**

**Supplement Table 2 Summary of study assessments and timelines**

| Time point | Measurement | Post-allocation | | | | | Mode of Administration |
| --- | --- | --- | --- | --- | --- | --- | --- |
|  |  | Baseline | Training sessions | 10 weeks assessments | | Follow-up 3 months |  |
|  |  | **T0** |  | **T1** | | **T2** |  |
| **Enrollment** | | | | | | | |
| **Eligibility screening** |  |  |  |  | |  | Paper and pencil |
| **Informed consent** |  |  |  |  | |  | Paper and pencil |
| **Screening** | **General information** | | | | | | |
|  | Demographic information | **√** |  |  | |  | Paper and pencil |
|  | **Daily functions** | | | | | | |
|  | Activities of Daily Living (ADL) | **√** |  |  | |  | Observation |
|  | **Optic examination** | | | | | | |
|  | Achromatopsia anomalous trichromatic test | **√** |  |  | |  | Paper and pencil |
|  | **Overall cognitive function** | | | | | | |
|  | Montreal cognitive assessment (MoCA) | **√** |  |  | |  | Paper and pencil |
|  | Mini-mental State Examination (MMSE) | **√** |  |  | |  | Paper and pencil |
| **Randomized allocation** | | |  |  |  | |  |
| **Intervention** | | |  |  |  | |  |
| **Training tasks** | | | | | | | |
| Warm-up exercise | **√** | | | | | | Paper and pencil |
| Practice in order | **√** | | | | | | Cylinder socket blocks |
| Expanding training | **√** | | | | | |  |
| **Assessments** | | | | | | | |
| **Primary objective** | **Executive function** | | | | | | |
|  | Trail Making Test A-B | **√** |  | **√** | | **√** | Paper and pencil |
|  | Color-Word Matching Stroop task | **√** |  | **√** | | **√** | Computer |
|  | **Psychomotor speed** | | | | | | |
|  | Finger Tapping test | **√** |  | **√** | | **√** | Device |
|  | Reaction Time test | **√** |  | **√** | | **√** | Device |
|  | **Working memory** | | | | | | |
|  | Digit span test | **√** |  | **√** | | **√** | Paper and pencil |
